# Performance of Firth-and ***logF***-type penalized methods in risk prediction for small or sparse binary data

**DOI:** 10.1186/s12874-017-0313-9

**Published:** 2017-02-23

**Authors:** M. Shafiqur Rahman, Mahbuba Sultana

**Affiliations:** Institute of Statistical Research and Training, University of Dhaka, Dhaka, Bangladesh

**Keywords:** Prediction model, Separation, Performance measures, Overfitting

## Abstract

**Background:**

When developing risk models for binary data with small or sparse data sets, the standard maximum likelihood estimation (MLE) based logistic regression faces several problems including biased or infinite estimate of the regression coefficient and frequent convergence failure of the likelihood due to separation. The problem of separation occurs commonly even if sample size is large but there is sufficient number of strong predictors. In the presence of separation, even if one develops the model, it produces overfitted model with poor predictive performance. Firth-and log*F*-type penalized regression methods are popular alternative to MLE, particularly for solving separation-problem. Despite the attractive advantages, their use in risk prediction is very limited. This paper evaluated these methods in risk prediction in comparison with MLE and other commonly used penalized methods such as ridge.

**Methods:**

The predictive performance of the methods was evaluated through assessing calibration, discrimination and overall predictive performance using an extensive simulation study. Further an illustration of the methods were provided using a real data example with low prevalence of outcome.

**Results:**

The MLE showed poor performance in risk prediction in small or sparse data sets. All penalized methods offered some improvements in calibration, discrimination and overall predictive performance. Although the Firth-and log*F*-type methods showed almost equal amount of improvement, Firth-type penalization produces some bias in the average predicted probability, and the amount of bias is even larger than that produced by MLE. Of the log*F*(1,1) and log*F*(2,2) penalization, log*F*(2,2) provides slight bias in the estimate of regression coefficient of binary predictor and log*F*(1,1) performed better in all aspects. Similarly, ridge performed well in discrimination and overall predictive performance but it often produces underfitted model and has high rate of convergence failure (even the rate is higher than that for MLE), probably due to the separation problem.

**Conclusions:**

The log*F*-type penalized method, particularly log*F*(1,1) could be used in practice when developing risk model for small or sparse data sets.

## Background

In many areas of clinical research, risk models for binary data are usually developed in the maximum-likelihood (ML) based logistic regression framework to predict the risk of a patient’s future health status such as death or illness [1, 2]. For example, in cardiology, models may be developed to predict the risk of having cardiovascular disease. Predictions based on these models are useful to both doctor and patient in making joint decision on future course of treatment. However, before using these models in risk prediction it is essential to assess their predictive performance using data other than that used to develop the models, which is termed as ‘validation’ [3, 4]. A good risk model is expected to demonstrate good calibration (accuracy of prediction) and discrimination (the ability of model to distinguish between low-and-high risk patients) in new dataset. A risk model that perform well in development data (that used to fit the model called ‘training’ set) may not perform similar to the validation data (that used to validate the model called ‘test’ set). One of the main reasons for not performing well in test data is model overfitting which causes too high prediction for high risk patients and too low for low risk patients. The overfitting occurs frequently when the number of events in training data is lower than the number of risk factors. After employing expert knowledge even if one fits the model with reduced the number of predictors, the ratio of the number of event to the number of predictors (EPV) often very low. However, as a rule of thumb, it has been suggested in literature that the risk model performs well when EPV is at least 10 [5]. Although the choice of this cut-off has some criticisms [6] for not being based on scientific reasoning except empirical evidence, it is found useful for quantifying the amount of information in the data relative to model complexity [7, 8]. However, the requirement of minimum EPV is often difficult to achieve when the risk models develop for low-dimensional data with rare outcome or small-and moderate-size, and for high-dimensional data where the number of predictors is usually higher than the number of sample observations.

To overcome the problem related to overfitting, some studies [9, 10] explored the use of penalized regression methods in risk prediction. Of them Ambler et al. [9] explored the use of two popular penalized regression methods, such as ridge [11] and lasso [12], in risk prediction for low-dimensional survival data with rare events and found that both methods improve calibration and discrimination compared with the ML based standard Cox models. Pavlou et al. [10] reviewed and evaluated ridge and lasso and their some extensions, such as elastic net, adaptive lasso etc [13–15], in risk prediction for low dimensional binary data with rare events and found that these methods can offers improvement, particularly for model overfitting, over the standard logistic regression model. Although these studies showed some improvement in risk prediction for rare-event data by using the penalized methods, there is no specific guidelines how risk prediction can be managed in the presence of separation, which frequently occur for such rare-event or sparse data. More specifically, the problem of separation, first reported by Albert and Anderson [16], is the case where one or more predictors have strong effects on response and hence (nearly) perfectly predict the outcome of interest. Table 1 presents an example of both complete (perfect prediction) and quazi-complete separation (nearly perfect prediction) caused by a dichotomous predictor *X* against binary outcome *Y*.

**Table 1:** Example of separation due to a dichotomous predictor *X* against outcome *Y*

Complete separation				Quazi-complete separation			
Y				Y			
		1	0			1	0
X	A	10	0	X	A	10	0
	B	0	10		B	2	8

Number in each cell indicates number of observations

Separation may occur even if the data is large but there is sufficient number of strong predictors. The likelihood of separation is higher for categorical predictors with rare category compared to the continuous predictor [17]. When developing model in the presence of separation, ML based logistic regression faces several problems [16, 18]. These includes lack of convergence of maximum likelihood and even if it converges it produces biased (sometimes infinite) estimate of the regression coefficient [17]. An alternative to the ML approach in this situation is Firth’s penalized method [19]. This approach removes the first order term (*O*(*n* ^−1^)) in the asymptotic bias expansion of the MLEs of the regression parameters by modifying the score equation with a penalty terms known as Jeffreys invariant prior. Heinze and Schemper [17] provided an application of Firth’s method to the solution of the problem of separation in the logistic regression. Further the applications of Firth’s method have been provided to proportional and conditional logistic regressions for situations with small-sample bias reduction and solution to problem of separation [20, 21].

However, one of the criticisms of Firth-type penalty in recent studies [22, 23] is that it depends on observed covariate data which can lead to artifacts such as estimates lying outside the range of prior median and the MLE (which is known as Bayesian non-collapsibility). An alternative to this, Greenland and Mansournia [22, 23] suggested log*F*(1,1) and log*F*(2,2) priors as default prior for logistic regression. As argued by the authors, the proposed log*F* priors are transparent, computationally simple, and reasonable for logistic regression. However, despite the attractive advantages of these penalized methods including Firth’s method for sparse or small data sets, limited studies have been conducted to explore their use in risk prediction. This paper evaluates the predictive performance of these penalized methods for sparse data and compares the results with the ML based method and the other commonly used penalized method such as ridge. Although lasso is a commonly used method, it is popular for variable selection. Risk prediction and variable selection are different issues, and in this paper we have focused on prediction and hence excluded lasso.

This paper is organized as follows. The next section briefly describes all penalized methods under study. Then the following sections describe the simulation study, an illustration of the methodologies using stress ecocardiography data, and finally discussion and conclusions.

## Methods

### Maximum likelihood based logistic regression model

Let *Y*_*i*_,(*i*=1,2,…,*n*), be a binary outcome (0/1) for the *i*th subject which follows Bernoulli distribution with the probability *π*_*i*_=Pr(*Y*_*i*_=1). The logistic regression model can be defined as

$$\begin{array}{*{20}l} \text{logit}[\pi_{i}|\boldsymbol{x}_{i})]=\eta_{i}=\boldsymbol{\beta}^{T}\boldsymbol{x}_{i}, \end{array} $$

where ***β*** ^*T*^ is a vector of regression coefficients of length (*k*+1), and ***x***_*i*_ is the *i*th row vector of the predictor matrix ***x*** which has order *n*×(*k*+1). The term *η*_*i*_=***β*** ^*T*^ ***x***_*i*_ is called as risk score or ‘prognostic index’.

In standard MLE, the model is fitted by maximizing the log likelihood denoted by *l*(***β***).

### Penalized methods for logistic regression model

Whereas in penalized methods, *l*(***β***) is maximized subject to constraints on the values of regression coefficients. The constraints are fixed in such a way so that the regression coefficient shrinks towards zero in comparison with MLE, which may help to alleviate overfitting. More specifically, the penalized regression coefficient is obtained by maximizing the penalized log likelihood denoted by ł (***β***)−pen(***β***), where pen(***β***) is the ‘penalty term’. The penalty term is the functional form of constraints. The penalized methods differ from each others in the choice of penalty term. The following subsection briefly discusses some popular penalized methods.

#### Firth’s penalized method

In order to remove first order bias in MLEs of the regression coefficient, Firth [19] suggested to use penalty term $\frac {1}{2}\text {trace}[I(\boldsymbol {\beta })^{-1}\partial I(\boldsymbol {\beta })/\partial \beta _{j}]$ in the ML based score equation *U*(*β*_*j*_)=*∂* *l*(***β***)/*∂* *β*_*j*_=0. The modified score equations are then *U*(*β*_*j*_)^∗^=*U*(*β*_*j*_)+1/2trace[*I*(***β***)^−1^ *∂* *I*(***β***)/*∂* *β*_*j*_]=0 (*j*=1,…,*k*), where *I*(***β***)^−1^ is the inverse of information matrix evaluated at ***β***. The corresponding penalized log-likelihood function for the above modified score function is *l*(***β***)+1/2 log|*I*(***β***)|. The penalty term used above is known as Jeffreys invariant prior and its influence is asymptotically negligible. The Firth type penalized MLE of ***β*** is thus $\hat {\boldsymbol {\beta }}=\text {argmax}\left \{ l(\boldsymbol {\beta })+1/2 \log |I(\boldsymbol {\beta })|\right \}$. This approach is known as bias preventive rather than corrective. However, Greenland and Mansournia [23] identified some problems in Jeffreys prior (equivalent to Firth’s penalty term). These includes i)Jeffrey’s prior is data-dependent and includes correlation between covariates ii) the marginal prior for a given *β* can change in opaque ways as model covariates are added or deleted, which may provide surprising results in sparse dataset, and iii) it is not clear how the penalty translate into prior probabilities for odds ratios.

#### Penalized method based on ***logF*** prior

To overcome these problems, Greenland and Mansournia [23] proposed a class of penalty functions pen(***β***)= ln(|*I*(***β***)|^−*m*^) indexed by *m*≥0, which produce MLE for *m*=0. Then the penalized log-likelihood is equal to *l*(***β***)+*m* ***β***/2−*m* ln(1+*e* ^***β***^). They showed that the antilog of the penalty term *m* ***β***/2−*m* ln(1+*e* ^***β***^) is proportional to a log*F*(*m*,*m*) density for ***β***, which is the conjugate family for binomial logistic regression [24, 25]. It is noted that the prior degrees of freedom *m* in log*F* prior is exactly the number of observations added by the prior. Then the corresponding penalized ML estimate can be obtained as $\hat {\boldsymbol {\beta }}=\text {argmax}\left \{l(\boldsymbol {\beta })+m\boldsymbol {\beta }/2-m\ln (1+e^{\boldsymbol {\beta }}) \right \}$. This shows that $\hat {\boldsymbol {\beta }}$ has first order (*O*(*n* ^−1^)) bias of zero for *m*=1, away from zero for *m*<1, and shrinks toward zero for *m*>1. This showed that *F*(0,0) is equivalent to MLE, and *F*(1,1) includes Jefrreys prior in one parameter model, for example, matched pair case-control. Greenland and Mansournia strongly argued against imposing a prior on the intercept to make sure that the mean predicted probability of binary condition is equal to the proportion of events. In this study, we focused on *F*(1,1) and *F*(2,2) prior for computational simplicity.

#### Ridge penalized method

Le Cessie and van Houwelingen [11] uses the penalty term as $\lambda _{2}\sum _{j=1}^{k} \beta _{j}^{2}$, where *λ*_2_ is a tuning parameter that modulates the trade-off between the likelihood term and the penalty term and is usually selected as data-driven procedure such as cross validation. The ridge log-likelihood is thus defined as $l(\boldsymbol {\beta })-\lambda _{2}\sum _{j=1}^{k}\beta _{j}^{2}$ and hence $\hat {\boldsymbol {\beta }}=\text {argmax}\left \{ l(\boldsymbol {\beta })-\lambda _{2}\sum _{j=1}^{k}\beta _{j}^{2} \right \}$. Ridge was initially developed to solve the problems due to multicolinearity. However, it shrinks the regression coefficient towards nearly zero and hence can be performed well to alleviate overfitting in risk prediction in the scenario with correlated predictors.

### Evaluating predictive performance

Three common approaches to evaluate the predictive performance of a risk model [26]. These are i) calibration (the agreement between the observed and predicted risk in a group of subjects) ii) discrimination (the ability of model to distinguish between low-and high-risk patients) iii) overall prediction accuracy.

**Calibration:** We assessed calibration by calculating calibration slope, which can be obtained by re-fitting a binary logistic regression model with linear predictor or prognostic index (PI) derived from the original model as the only predictor. The estimated slope $\hat {\beta }_{PI}$ is the calibration slope. If $\hat {\beta }_{PI}=1$, it suggests perfect calibration; $\hat {\beta }_{PI}<1$ suggests overfitting, and $\hat {\beta }_{PI}>1$ suggest underfitting.

**Discrimination:** We assessed discriminating ability of the model by quantifying the area under receiver operating characteristic curve (AUC), graph of sensitivity (true-positive rate) versus one minus specificity (true-negative rate) evaluated at consecutive threshold values of the predicted risk score or probability derived from the model. Alternatively AUC can be obtained by quantifying the probability that, for a randomly selected pair of subjects, the subject who experienced the event of interest had higher predicted risk derived from the model than those without experiencing the event. A value AUC = 0.5 indicates no discrimination and 1 suggest perfect discrimination.

**Overall predictive performance:** The overall prediction accuracy is quantified using Brier score, which is the mean of the squared difference between the observed and predicted risk for each patient derived from the model. The lower the BS, the better the prediction of a model and BS=0 indicates perfect prediction. For ease of interpretation we reported root BS(rBS). In addition to the rBS, we also reported average predictive probability (APP) of the model to see how the predicted value differ from the corresponding observed value.

### Software

All the analyses and simulations were conducted in Stata version 12. Several Stata packages and functions were used to fit the models in different methods under study. These includes ‘logit’, ‘firthlogit’, ‘penlogit’, and ‘plogit’ along with ‘plsearch’ for MLE, FIRTH, log*F* and RIDGE, respectively. The calculation of calibration slope and Brier score were performed using self written Stata code and AUC using the package ‘roctab’.

## Results

### Example data: stress echocardiography data

The dataset used for simulation and illustration is in public domain and originally extracted from the study conducted by Krivokapich et al. [27] where the aim was to quantify the prognostic value of dobutamine stress echocardiography (DSE) in predicting cardiac events in 558 patients (male 220 and female 338) with known or suspected coronary artery disease. The responses of interest whether or not a patient suffered from either of ‘death due to cardiac arrest’, or ‘ myocardial infarction (MI)’, or ‘ revascularization by percutaneous transluminal coronary angioplasty (PTCA)’ or ‘coronary artery bypass grafting surgery (CABG)’ over the next year after having the test. There were 24 patients with cardiac death, 28 with MI, 27 with PTCA, 33 with CABG and 89 with any cardiac event (Cevent), which implies that the each of the events was rare. The main predictor of interest are age, history of hypertension (HT: yes/no) and diabetics mellitus (DM: yes/no), history of prior MI (yes/no) and PTCA (yes/no), status of DSE test (positive DSE:positive/negative), wall motion anamoly on echocardiogram (rest WMA:yes/no), ejection fraction on dobutamine(Dobutamine EF), and base ejection fraction (base EF).

### Simulation study

The performance of the penalized methods in risk prediction over standard ML based logistic regression were investigated using a simulation study. We conducted simulation i) firstly to assess and compare the properties of the regression coefficients of the different methods (MLE, FIRTH, log*F*(1,1), log*F*(2,2), RIDGE) under study and ii) secondly to assess and compare the predictive performance between the methods.

#### Assessing the properties of the regression coefficients

To assess the properties of the regression coefficients such as bias and mean squared error (MSE), we generated two independent predictors of which one is continuous (*X*_1_) generated from standard normal and the other is dichotomous (*X*_2_) generated from Bernoulli distribution with 50% events. We then generated binary response from Bernoulli distribution with probability *π*_*i*_ (*i*=1,…,*n*) calculated from true logistic model logit(*π*)=*β*_0_+*β*_1_ *X*_1_+*β*_2_ *X*_2_, where *β*_1_=0.30 and *β*_2_=0.9. With this combination, the binary covariate created separation for some of the simulated datasets particularly with low prevalence. The value of *β*_0_ vary to generate data with varying level of prevalence. The scenarios were created by varying the prevalence, on an average, (*p*) as 5.5, 11.5 20.4 and 39.6% for a fixed sample size *n*=120. For each scenario, 1000 datasets were generated and all regression approaches under study were fitted to each dataset. When fitting RIDGE the respective tuning parameters were selected through 10-fold cross validation. The estimates of the regression coefficients of the respective models were obtained as the mean over the number of simulations where convergence achieved. Noted that only MLE and RIDGE were failed to converge (due to low prevalence or separation or both) in some datasets, and the maximum failure rate for MLE and RIDGE were 13 and 51%, respectively for the lowest prevalence scenario. The failure rate decreases as the prevalence increases. Finally the relative bias (%) and mean squared error (MSE) of the estimates were reported and compared if the performance vary across the scenarios.

The results in Table 2 showed that the RIDGE estimator, in general, provides the highest amount of relative bias (%), which is followed by the MLE and log*F*(2,2) whereas FIRTH and log*F*(2,2) provides negligible bias. For the coefficient of the dichotomous predictor (*β*_2_) log*F*(2,2) provides more bias compared to those for continuous predictor (*β*_1_). The amount of bias, in general, is the highest for the low prevalence data and the lowest for the high prevalence data. However, the RIDGE, in general, produces the lowest MSE, and the highest MSE is produced by the MLE for *β*_1_ and by FIRTH for *β*_2_. The amount of MSE, in general, decreases with the increasing prevalence.

**Table 2:** Performance of the estimated regression coefficients of models fitted using different methods under study

		Estimates					Relative bias (%)					MSE				
Coefficient	Prev.(%)	MLE	FIRTH	log*F*(1,1)	log*F*(2,2)	RIDGE	MLE	FIRTH	log*F*(1,1)	log*F*(2,2)	RIDGE	MLE	FIRTH	log*F*(1,1)	log*F*(2,2)	RIDGE
	5.5	0.33	0.29	0.30	0.28	0.26	10.84	-3.56	-1.29	-6.82	-14.72	0.25	0.19	0.20	0.18	0.10
	11.5	0.33	0.31	0.32	0.31	0.26	8.71	2.68	5.46	2.44	-12.23	0.10	0.09	0.10	0.09	0.07
*β*_1_	20.4	0.30	0.29	0.29	0.29	0.24	0.14	-4.54	-1.81	-3.62	-19.50	0.07	0.06	0.06	0.06	0.05
	39.6	0.31	0.30	0.31	0.30	0.25	4.13	-0.14	2.62	1.19	-16.32	0.04	0.04	0.04	0.04	0.03
	59.9	0.31	0.29	0.30	0.30	0.25	1.68	-2.48	0.20	-1.19	-16.87	0.05	0.04	0.04	0.04	0.04
	5.5	0.80	0.87	0.86	0.71	0.58	-11.19	-3.41	-4.19	-21.26	-35.13	0.66	0.76	0.75	0.50	0.36
	11.5	0.98	0.91	0.91	0.82	0.78	8.92	1.50	0.67	-8.85	-13.36	0.48	0.42	0.42	0.33	0.29
*β*_2_	20.4	0.95	0.90	0.89	0.84	0.76	5.34	0.02	-0.74	-6.12	-15.61	0.27	0.23	0.23	0.21	0.23
	39.6	0.92	0.89	0.89	0.86	0.75	2.19	-0.74	-1.50	-4.94	-16.14	0.18	0.16	0.16	0.15	0.18
	59.9	0.92	0.89	0.89	0.86	0.75	2.36	-0.56	-1.33	-4.77	-16.36	0.15	0.14	0.14	0.13	0.17

Relative bias and MSE were calculated over number of simulations for which the convergence is achieved. The maximum failure rate of convergence, out of 1000 simulations, for MLE was 13% for lowest prevalence, and for RIDGE it is 51%. Max MCE=0.0251

#### Assessing the predictive performance

To assess the predictive performance of the methods, we conducted two simulation series following the simulation design in Pavlou et al. [10] used for similar type of study. The first simulation series is based on the real stress echocardiography data where only responses were generated and in the second simulation series we generated both covariates and responses.

#### Stress echocardiography simulation

In the first simulation series based on real data, we simulated data and evaluated the predictive performance of the models for different EPV scenarios using the following steps:

- Fit the following logistic regression model for the response “any cardiac event” with Firth’s penalized method (to avoid bias in the estimate of the regression coefficient) to obtain the true model:
  $$\begin{array}{*{20}l} {}\text{logit(Pr(Cevent=1))}&=\beta_{0}+\beta_{1}{dobef}+ \beta_{2} wma +\beta_{3} posse\\&\quad+\beta_{4} bsef+\beta_{5} ht+\beta_{6} age \end{array} $$
- To create a training data, choose the EPV and prevalence (*prev*), and then calculate sample size for the respective EPV given the number of predictors *p* as $n=\frac {EPV \times p}{prev}$. Sample with replacement the *n* values of the covariates in the true model from original data. For each of the *n* values of the covariates, simulate new responses from Bernoulli distribution with the probability calculated from the fitted model. However, replace the value of *β*_0_ by -0.65 to confirm the prevalence of the response (*prev*), on an average, 15.5% for all EPV scenarios.
- With this combination, check and record if separation occurred due to any of the binary covariates (‘posse’ or ‘wma’, or ‘ht’ or combination of them). Otherwise to create separation, enlarge the true value of the respective coefficient of the binary covariate to some extents. Note that the chances of separation is expected to increase with decreasing EPV value.
- To create a test dataset, sample with replacement *m*×*n* (*m* times of the original data of size *n*=558, we considered *m*=2) values of the covariates. Then simulate the corresponding new responses from the same true model used for simulating training data.
- Repeat the steps (ii)-(iii) to produce 1000 training and 1000 test datasets.
- Fit the risk models (using MLE and all types of penalized regression methods under study) to each of the training data sets and check whether convergence was achieved. Then evaluate their predictive performance (if convergence achieved) by means of calibration slope, AUC, root Brier score, and average predictive probability (APP) using the corresponding test dataset. Summarize the predictive performance over the number of simulations for which convergence is achieved.

**Fig. 1:**
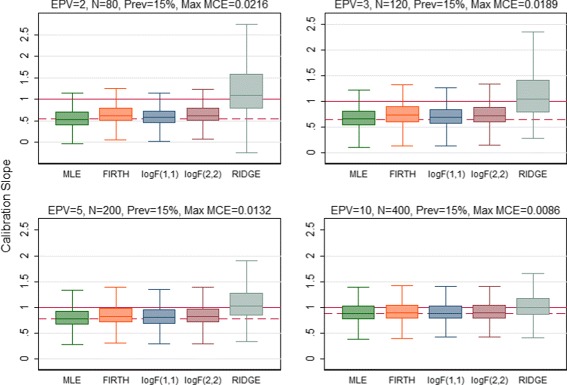
Performance of the methods was assessed using calibration slope and compared. Results were summarized over the number of simulations for which convergence is achieved. The maximum failure rate of convergence for RIDGE, out of 1000 simulations, is 8.4% when EPV=2. The values outside the whisker were not plotted to make the plot readable. The *horizontal dash line* is the median calibration slope for MLE and the *solide line* is the optimal value

**Fig. 2:**
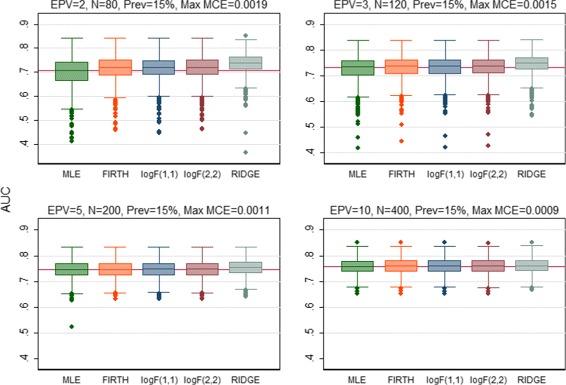
Performance of the methods was assessed using area under ROC (AUC) and compared. Results were summarized over the number of simulations for which convergence is achieved. The maximum failure rate of convergence for RIDGE, out of 1000 simulations, is 8.4% when EPV=2. The *horizontal solide line* is the median AUC for MLE

**Fig. 3:**
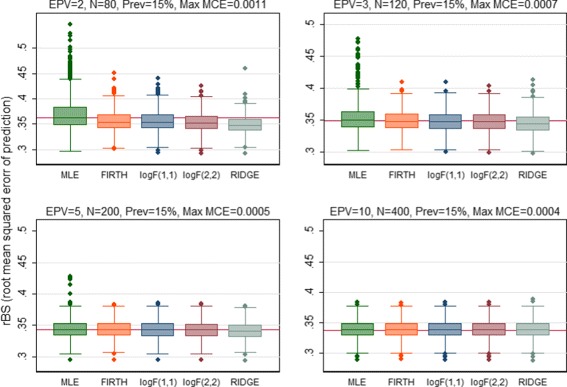
Performance of the methods was assessed using root Brier score (rBS) and compared. Results were summarized over the number of simulations for which convergence is achieved. The maximum failure rate of convergence for RIDGE, out of 1000 simulations, is 8.4% when EPV=2. The *horizontal solide line* is the median rBS for MLE

**Fig. 4:**
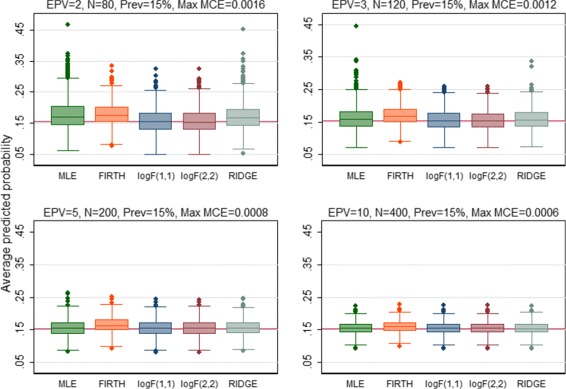
Performance of the methods was assessed using Average Predicted Probability (APP) and compared. Results were summarized over the number of simulations for which convergence is achieved. The maximum failure rate of convergence for RIDGE, out of 1000 simulations, is 8.4% when EPV=2. The *horizontal solide line* is the observed APP

The predictive performance of all regression methods was investigated against EPV=2, 3, 5, 10 to see if the performance vary across the scenarios. When the predictive performance against EPV was assessed by means of calibration slope, the MLE showed poor performance by producing overfitted model (calibration slope substantially lower than 1) for EPV=2, 3, 5 (Fig. 1). All penalized methods offered improvement to some extents except the RIDGE which produced underfitted model (the average value of the calibration slope greater than 1 with high SD). In addition, the RIDGE failed to converge for the maximum 8.4% of the simulations particularly when EPV=2. Almost equal improvement was offered by the Firth-type and both the log*F*(1,1) and log*F*(2,2) penalized methods. In general all methods including MLE showed almost equal performance in terms of calibration for high EPV (EPV=10). When the predictive performance (discriminatory ability) was assessed through AUC, all penalized methods showed better performance with greater AUC than MLE for the low EPV scenarios (Fig. 2). Of them the RIDGE provided highest AUC value. However, the amount of improvement in the discrimination, in general, was comparatively lower than that for calibration. All methods perform almost equally for high EPV (EPV=10). Similarly the penalized methods offered improvement in the overall predictive performance for individual prediction assessed through rBS to some extents for low EPV (Fig. 3). Of them, the RIDGE offered greater improvement. However, for low EPV while both the log*F*(1,1) and log*F*(2,2) penalized methods provided accurate estimate of the true average predicted probability (APP) 15.2%, the FIRTH-type penalized method overestimate the true value. The amount of bias in FIRTH-type estimate is even larger than that produced by MLE and RIDGE (Fig. 4).

#### Further simulation

In the second simulation series with the same EPV scenarios, we simulated both covariates and response under two predictive models, one with weak predictive ability and the other with strong predictive ability, using the following steps:

- For creating training data, choose the EPV and prevalence (*prev*) and calculate sample size (*n*) for the given EPV value and the number of predictors using the same formula previously used.
- For each observation in training data, first simulate three continuous predictors (*X*_1_,*X*_2_,*X*_3_) independently from standard normal distribution and two binary predictors (*X*_4_,*X*_5_) independently from Bernoulli distribution one with low (20%) and the other with high (60%) prevalence.
- Simulate the corresponding responses from Bernoulli dis-tribution with probability calculated from the true model:
  $$\begin{array}{*{20}l}{}\text{logit}(\pi)=\beta_{0}+\beta_{1} X_{1} +\beta_{2} X_{2}+ \beta_{3} X_{3}+\beta_{4} X_{4}+\beta_{5}X_{5}. \end{array} $$
  For the model with weak predictive ability, the values of the regression coefficient were set as *β*_0_=−1.5*β*_1_=0.2,*β*_2_=0.5*β*_3_=−0.03,*β*_4_=0.05 and *β*_5_=−0.6 and for the model with strong predictive ability, the corresponding true values were set as *β*_0_=−3.5,*β*_1_=1.2,*β*_2_=−0.9*β*_3_=0.9,*β*_4_=1.2 and *β*_5_=1.2. In each case, the value of the *β*_0_ confirms the desired prevalence of the response. With this combination, the binary covarites *X*_5_ in the model with weak predictive ability and *X*_4_ in the model with strong predictive ability create separation in some of the simulations. Check and record if separation occurred.
- Create test data with size 1000 (much larger than the training data) for the similar level of EPV and prevalence. For each observation in the test data, simulate the same predictors as in the test data and the corresponding response from the same true model.
- Repeat the steps (ii)-(iv) to produce 1000 training and 1000 test datasets
- Fit risk models (using all methods) using training data, count if convergence was achieved for the respective model, and evaluate their predictive performance (if convergence was achieved in training data) using test data as before. Finally summarize the predictive performance over the number of simulations for which convergence is achieved.

The results revealed that, for both predictive models (weak and strong predictive abilities), all the penalized methods offered improvement in calibration over MLE for low EPV, except for the RIDGE which in turn provided underfitted model (calibration slope grater than 1 with high SD) (Table 3). The amount of improvement by the other penalized methods was almost equal. However, all the penalized methods except the RIDGE offered negligible improvement in the discrimination for low EPV. Similarly all the penalized methods showed improvement to some extents in the overall predictive performance by lowering the rBS value compared to that for MLE. For both predictive model, the average predicted probability (APP) estimated by the both the log*F*(1,1) and log*F*(2,2) were almost equal to the average observed probability, however the Firth-type penalized method introduced positive bias in the estimate of the average probability. The amount of bias was even larger than that for MLE and RIDGE. In case of both models, the maximum failure of convergence (due to separation or low EPV or both) was reported for RIDGE.

**Table 3:** Performance measures for the model s with both weak and strong predictive ability. Results were summarized over the number of simulations for which convergence is achieved. The maximum failure rate of convergence for RIDGE with weak predictive ability, out of 1000 simulations, is 40% for the lowest EPV

Model with weak predictive ability											
		Calibration slope, Max MCE=0.0235					AUC, Max MCE=0.0012				
EPV (N)		MLE	FIRTH	log*F*(1,1)	log*F*(2,2)	RIDGE	MLE	FIRTH	log*F*(1,1)	log*F*(2,2)	RIDGE
2(67)	Mean	0.367	0.414	0.383	0.424	1.029	0.606	0.605	0.605	0.607	0.628
	SD	0.277	0.303	0.281	0.302	0.847	0.060	0.058	0.059	0.059	0.042
3(100)	Mean	0.472	0.512	0.487	0.517	1.027	0.613	0.613	0.613	0.614	0.626
	SD	0.305	0.326	0.311	0.324	0.757	0.054	0.054	0.054	0.054	0.041
5(167)	Mean	0.621	0.658	0.637	0.658	1.055	0.629	0.630	0.630	0.630	0.635
	SD	0.317	0.328	0.317	0.323	0.667	0.046	0.046	0.046	0.046	0.039
10(334)	Mean	0.797	0.814	0.801	0.812	1.076	0.645	0.645	0.645	0.646	0.646
	SD	0.286	0.289	0.282	0.286	0.504	0.037	0.037	0.037	0.037	0.035
		root Brier Score, Max MCE=0.0007					APP (True 0.152), Max MCE=0.0015				
EPV(N)		MLE	FIRTH	log*F*(1,1)	log*F*(2,2)	RIDGE	MLE	FIRTH	log*F*(1,1)	log*F*(2,2)	RIDGE
2(67)	Mean	0.370	0.369	0.367	0.365	0.360	0.159	0.178	0.154	0.153	0.156
	SD	0.022	0.019	0.019	0.018	0.017	0.045	0.041	0.044	0.044	0.044
3(100)	Mean	0.363	0.362	0.361	0.360	0.358	0.156	0.171	0.154	0.154	0.155
	SD	0.018	0.017	0.017	0.017	0.016	0.035	0.033	0.035	0.035	0.035
5 (167)	Mean	0.357	0.357	0.357	0.356	0.355	0.153	0.163	0.153	0.153	0.152
	SD	0.017	0.016	0.017	0.016	0.016	0.028	0.027	0.027	0.027	0.027
10 (334)	Mean	0.354	0.354	0.354	0.354	0.354	0.151	0.157	0.151	0.151	0.151
	SD	0.016	0.015	0.016	0.016	0.015	0.020	0.019	0.020	0.020	0.019
Model with strong predictive ability											
		Calibration slope, Max MCE=0.0344					AUC, Max MCE=0.0024				
EPV (N)		MLE	FIRTH	log*F*(1,1)	log*F*(2,2)	RIDGE	MLE	FIRTH	log*F*(1,1)	log*F*(2,2)	RIDGE
2(67)	Mean	0.659	0.825	0.784	0.890	1.252	0.831	0.831	0.832	0.834	0.832
	SD	0.296	0.310	0.268	0.273	0.742	0.039	0.039	0.038	0.037	0.037
3 (100)	Mean	0.774	0.888	0.857	0.931	1.125	0.845	0.845	0.846	0.846	0.845
	SD	0.236	0.251	0.231	0.233	0.292	0.028	0.028	0.028	0.028	0.028
5(167)	Mean	0.868	0.934	0.917	0.963	1.066	0.854	0.854	0.854	0.855	0.854
	SD	0.218	0.226	0.216	0.217	0.224	0.024	0.023	0.023	0.023	0.023
10(334)	Mean	0.933	0.959	0.955	0.979	1.016	0.860	0.860	0.860	0.860	0.860
	SD	0.167	0.169	0.166	0.167	0.159	0.022	0.022	0.022	0.022	0.022
		root Brier Score, Max MCE=0.0009					APP (True 0.162), Max MCE=0.0014				
EPV (N)		MLE	FIRTH	log*F*(1,1)	log*F*(2,2)	RIDGE	MLE	FIRTH	log*F*(1,1)	log*F*(2,2)	RIDGE
2 (67)	Mean	0.338	0.331	0.330	0.327	0.328	0.172	0.182	0.164	0.163	0.167
	SD	0.030	0.022	0.021	0.020	0.019	0.045	0.040	0.042	0.042	0.042
3(100)	Mean	0.323	0.321	0.321	0.320	0.320	0.165	0.175	0.163	0.163	0.164
	SD	0.018	0.016	0.017	0.016	0.016	0.033	0.032	0.033	0.033	0.032
5(167)	Mean	0.316	0.315	0.315	0.315	0.315	0.163	0.170	0.163	0.163	0.163
	SD	0.016	0.015	0.015	0.015	0.015	0.026	0.025	0.025	0.025	0.025
10(334)	Mean	0.310	0.310	0.310	0.310	0.310	0.163	0.166	0.163	0.163	0.163
	SD	0.016	0.015	0.015	0.015	0.015	0.019	0.019	0.019	0.019	0.019

*APP: Average Predicted Probability*

### Illustration using stress echocardiography data

The aim is to derive risk models using different penalized methods discussed earlier and the standard MLE to predict the risk of having a cardiac event and then to evaluate and compare their predictive performance. We fitted separate models for predicting the risk of each of the four cardiac events and a model for the risk of any of the events using each regression approaches; that is, a total of five models for each of the binary events were fitted using six different regression methods under study and altogether 25 models for all five binary responses.

The models were fitted using training data (contains 60% of total data randomly selected) and their predictive performance were evaluated using test data (contains rest of 40%). The associated predictors for each cardiac event were selected based on the information from literature and results of likelihood ratio test (LRT). Different combinations of predictors were tested using LRT to come up with a final model for each cardiac event. Then the same model was then fitted in training data using six different methods. Note that quasi-complete separation due to binary predictors in training data was identified for the responses ‘PTCA’ and ‘ cardiac death’, and hence, in case of convergence failure for RIDGE or MLE, the estimates reported are based on the last iteration. The estimated coefficients of the respective model are then summarized in Table 4. For all types of response, the estimated regression coefficients for MLE is larger than all penalized methods. Because all the methods shrink the coefficient towards zero. The amount of shrinking was higher for the RIDGE in the most of the cases. However, the main purpose here is to evaluate the predictive performance of the methods rather than comparing their estimated regression coefficients. The predictive performance of all models were then evaluated using test data, and the results were summarized in Table 5.

**Table 4:** Modeling the risk of cardiac events. Estimate of the regression coefficients with SE in the parenthesis

	Modeling the risk of MI				
	MLE	FIRTH	log*F*(1,1)	log*F*(2,2)	RIDGE
Dobutamine EF	-0.0503	-0.0492	-0.0508	-0.0513	-0.0413
	(0.0183)	(0.0178)	(0.0183)	(0.0182)	(0.0161)
Positive DSE	1.272	1.241	1.185	1.109	0.994
	(0.549)	(0.531)	(0.533)	(0.518)	(0.469)
Hist. of HT	1.115	0.923	0.973	0.866	0.657
	(0.789)	(0.716)	(0.716)	(0.662)	(0.542)
Intercept	-1.253	-1.028	-1.057	-0.901	-1.243
	(1.351)	(1.284)	(1.307)	(1.275)	(1.132)
	Modeling the risk of CABG				
Dobutamine EF	-0.0634	-0.0506	-0.0518	-0.0523	-0.0420
	(0.0181)	(0.0177)	(0.0181)	(0.0181)	(0.0161)
Positive DSE	1.568	1.190	1.137	1.068	0.971
	(0.551)	(0.529)	(0.531)	(0.516)	(0.468)
Intercept	0.272	-0.224	-0.206	-0.145	-0.683
	(1.122)	(1.120)	(1.140)	(1.131)	(1.029)
	Modeling the risk of PTCA				
Positive DSE	0.825	0.820	0.770	0.722	0.579
	(0.498)	(0.481)	(0.483)	(0.470)	(0.409)
Base EF	-0.0381	-0.0375	-0.0389	-0.0396	-0.0306
	(0.0204)	(0.0198)	(0.0202)	(0.0201)	(0.0168)
Hist. of MI	1.168	1.125	1.118	1.072	0.867
	(0.533)	(0.517)	(0.515)	(0.499)	(0.412)
Hist of PTCA	1.304	1.310	1.211	1.127	1.087
	(0.617)	(0.591)	(0.602)	(0.588)	(0.555)
Intercept	-1.754	-1.661	-1.646	-1.548	-1.809
	(1.184)	(1.151)	(1.167)	(1.153)	(0.968)
	Modeling the risk of cardiac death				
Positive DSE	1.084	1.061	1.026	0.974	0.873
	(0.489)	(0.474)	(0.478)	(0.467)	(0.436)
Hist. of DM	1.083	1.047	1.025	0.973	0.784
	(0.495)	(0.480)	(0.481)	(0.468)	(0.419)
Age	0.0347	0.0328	0.0344	0.0342	0.0229
	(0.0240)	(0.0236)	(0.0238)	(0.0236)	(0.0188)
Intercept	-6.040	-5.783	-5.965	-5.899	-4.960
	(1.787)	(1.750)	(1.769)	(1.753)	(1.379)
Positive DSE	1.064	1.045	1.047	0.989	0.940
	(0.332)	(0.327)	(0.327)	(0.264)	(0.248)
Dobutamine EF	-0.0381	-0.0372	-0.0384	-0.0364	-0.0333
	(0.0131)	(0.0128)	(0.0130)	(0.0103)	(0.00941)
	Modeling the risk of any cardiac event				
Rest WMA	-0.779	-0.758	-0.755	-0.839	-0.762
	(0.429)	(0.421)	(0.419)	(0.336)	(0.293)
Hist. of HT	0.823	0.781	0.794	0.728	0.632
	(0.393)	(0.384)	(0.384)	(0.302)	(0.276)
Intercept	0.107	0.115	0.151	0.0102	-0.0980
	(0.833)	(0.818)	(0.829)	(0.655)	(0.611)

**Table 5:** Performance of penalized methods in predicting cardiac events

Models for predicting the risk of MI (EPV ≈ 7)				
Methods	Calibration Slope	AUC	Brier Score	APP
MLE	0.696(0.258)	0.768(0.051)	0.047	0.051
Firth	0.706(0.260)	0.766(0.052)	0.049	0.057
log*F*(1,1)	0.713 (0.265)	0.769(0.051)	0.048	0.052
log*F*(2,2)	0.723 (0.271)	0.769(0.051)	0.048	0.052
RIDGE	0.772(0.309)	0.762(0.053)	0.047	0.050
Models for predicting the risk of CABG (EPV ≈ 10)				
MLE	0.912(0.219)	0.814(0.046)	0.057	0.056
Firth	0.909 (0.217)	0.814 (0.046)	0.056	0.059
log*F*(1,1)	0.921(0.221)	0.814(0.046)	0.056	0.055
log*F*(2,2)	0.926(0.223)	0.813(0.046)	0.057	0.055
RIDGE	0.886(0.217)	0.814(0.046)	0.057	0.055
Models for predicting the risk of PTCA (EPV ≈ 5)				
MLE	0.718 (0.291)	0.730(0.108)	0.034	0.061
Firth	0.721(0.279)	0.729(0.108)	0.035	0.066
log*F*(1,1)	0.721(0.298)	0.728(0.107)	0.034	0.061
log*F*(2,2)	0.720(0.305)	0.728(0.107)	0.034	0.061
RIDGE	0.774(0.544)	0.727(0.107)	0.033	0.061
Models for predicting the risk of cardiac death (EPV ≈ 6)				
MLE	0.661(0.529)	0.688(0.121)	0.024	0.062
Firth	0.680(0.545)	0.688 (0.121)	0.024	0.067
log*F*(1,1)	0.645(0.535)	0.687(0.120)	0.024	0.062
log*F*(2,2)	0.623 (0.538)	0.687 (0.120)	0.024	0.061
RIDGE	0.665 (0.608)	0.684 (0.121)	0.023	0.062
Models for predicting the risk of any cardiac event (EPV ≈ 15)				
MLE	0.942(0.206)	0.771(0.044)	0.059	0.164
Firth	0.946(0.207)	0.767 (0.044)	0.059	0.167
log*F*(1,1)	0.945(0.206)	0.770(0.044)	0.058	0.164
log*F*(2,2)	0.946(0.207)	0.770 (0.044)	0.058	0.164
RIDGE	1.004(0.222)	0.769(0.044)	0.056	0.165

Event Per Variable (EPV) was calculated based on the number of event in training data. Estimates of the performance measures with SE in the parenthesis

It is observed from results in Table 5 that all models faced the problem of overfitting (calibration slope <<1) particularly for those response for which the EPV is low (EPV <10). The amount of overfitting is lower for all penalized methods compared to MLE. In terms of discrimination all methods including MLE provided comparable results. For all types of response, the greater improvement was observed in the calibration (calibration slope) compared to those in both discrimination (AUC) and overall performance (BS). Firth methods produced higher value of the average predicted probability (APP) for all type of responses.

The probable reason for producing overfitted models (very low value of the calibration slope) even for the penalized methods is that the size of the test data and particularly the number of events for all types of response were very small compared to the number of events (approximately 100) required for correct estimation of the predictive accuracy measures [28]. Therefore, further the predictive performance of all models were evaluated in test data consisting of larger sample size and number of events compared to the previous test data. This was created by expanding 5 times the original (previous) test data so that the required number of events is achieved. In this procedure each subject replaced his/her information for the other 4 subjects. The results showed that calibration slope was comparatively more closer to 1 (suggesting improvement in calibration) for the penalized methods for all types of responses, particularly for which EPV was high (results not showed). Similar results were obtained for the AUC and Brier score for all types of models of all responses.

## Discussion

Penalized regression methods (such as RIDGE and LASSO) has increasingly being used for developing models for high dimensional data where the number of predictors is higher than the number of subjects. Furthermore several studies [29, 30] have also been conducted to make relative comparison between the methods for high dimensional case and found that RIDGE performed well when data have highly correlated predictors and LASSO performed well when variable selection is required. Although few studies [9, 10] evaluated RIDGE, LASSO and others in risk prediction for low-dimensional survival and binary data with few events, however, they often ignored Firth-and log*F*-type (such as log*F*(1,1) and log*F*(2,2)) penalized methods, despite their attractive advantages in reducing finite sample bias in the estimated regression coefficient and solving problem of separation that commonly occurs in low-dimensional small or sparse datasets. This paper explored the use of these methods in risk prediction for small and sparse data and compared their predictive performance with MLE and the other penalized method (RIDGE). In particular we focused on comparing the predictive performance of the methods through assessing calibration, discrimination and overall predictive performance when EPV is less than 10 in low-dimensional setting.

The results from simulation studies and illustration with real data revealed that while the MLE produced overfitted model with poor predictive performance (in terms of calibration), all penalized methods offered some improvements except for the RIDGE which in turn produced underfitted models (calibration slope greater than 1 with large variability). All other penalized methods (Firth-type and both log*F*(1,1) and log*F*(2,2)) offered similar amount of improvement in calibration. However, the improvement in the discrimination in general was lower than that in calibration. The reason can be explained similarly with Pavlou et al. [10] as that the penalized methods tend to shrink the predicted probability towards the average compared with the MLE and hence the ordering of the predicted probabilities with and without experiencing the event in most patient pairs tends to remain unchanged after shringkage, which resulted in small improvement in AUC values of the penalized methods over MLE. All the penalized methods offered some improvement in the overall predictive performance (lower BS compared to those with MLE). Although all penalized methods correctly estimate the average predicted probability, Firth-type penalization introduced bias. The findings are similar to what obtained in other studies [10] that explored the use of some penalized methods such as ridge, lasso etc in risk predictions for low-dimensional data.

## Conclusions

Based on the findings of the study it can be recommended to use log*F*-type penalized method instead of MLE in risk prediction for low dimensional data small or sparse data. Because firstly this approach showed minimum bias in the estimate of regression coefficient and greater improvement in predictive performance than MLE, particularly in calibration by removing the amount of overfitting to some extents. Secondly, this approach has some additional advantage particularly for solving the problems due to separation. Of the two types of log*F* penalization, log*F*(1,1) is preferable to log*F*(2,2) because log*F*(2,2) though provides similar predictive performance but produces some bias in the regression coefficient particularly for the dichotomous covariates. Although the Firth-type penalized method have great advantage for solving the problems related to separation and showed comparable results with the log*F*-type penalized methods with respect to calibration, discrimination and overall predictive performance, it produced bias in the estimate of the average predicted probability. The reason is that Firth’s approach imposes prior on the intercept (which control the average predicted probability) and as a result it shrink the average predicted probability towards 0.5 and hence produced upward bias in the average predicted probability. However, the log*F* make the intercept free from the penalization and hence correctly estimates the average predicted probability. Similarly although RIDGE showed greater improvement in the discrimination and the overall predictive performance, it often provides under-fitted model. The striking disadvantages of RIDGE is that it has frequent convergence failure for data with low EPV or if there is separation. The rate was high (even higher than MLE) if data have combination of both low EPV and separation. This finding is similar to those [31] which reported low EPV or separation or combination of both as one of the reasons for the convergence-failure in RIDGE, although other studies [32] reported it as wrong choice (small value) of tuning parameter.

In the presence of separation, developing a risk model using any other penalized methods, except for the Firth-type and log*F*-type methods, under study is challenging. Because RIDGE and LASSO-type penalized methods were originally developed particularly either for shrinking the regression coefficient or variable selection in high dimensional data rather than solving separation problem. However, the main limitation of log*F* type penalized approach is that it cannot be used directly for variable selection. If small-to moderate-level of variable selection is required in low-dimensional data with sufficient number of predictors, log*F* method can also be used in risk prediction after selecting important predictors using results from exploratory analysis of the data and likelihood ratio test conducted in different combinations of nested models.

This study did not focus on the use of Firth-type and log*F*-type penalized method in risk prediction for low-dimensional survival data with few events where standard Cox regression is reported to be unreliable [33]. Further research may be possible to evaluate the predictive performance of these methods in comparison with the standard Cox model and the other penalized methods.

## Abbreviations

APP: Average predicted probability
AUC: Area under receiver operating characteristic curve
EPV: Event per variable
FIRTH: Firth's penalized method
MLE: Maximum likelihood estimation
